# Psychosocial factors are preventive against coronary events in Japanese men with coronary artery disease: The Eastern Collaborative Group Study 7.7-year follow-up experience

**DOI:** 10.1186/s13030-015-0030-8

**Published:** 2015-01-17

**Authors:** Reiko Hori, Jun-ichiro Hayano, Kazuhiro Kimura, Nitaro Shibata, Fumio Kobayashi

**Affiliations:** Department of Health and Psychosocial Medicine, Aichi Medical University School of Medicine, 1-1 Yazako Karimata, Nagakute, Aichi 480-1195 Japan; Nagoya City University Graduate School of Medical Sciences and Medical School, Nagoya, Japan; Kimura Clinic, Kouka, Japan; Shinjyuku Mitui Clinic, Tokyo, Japan

**Keywords:** Coronary artery disease, Prognosis, Risk, Behavioural medicine, Psychosocial factor, Japanese

## Abstract

**Background:**

The Japanese Coronary-prone Behaviour Scale (JCBS) is a questionnaire developed by the Eastern Collaborative Group Study (ECGS), a multi-centre study of coronary-prone behaviour among Japanese men. Subscale C of the JCBS consists of 9 items that have been independently associated with the presence of coronary artery disease (CAD) in patients undergoing coronary angiography (CAG). There have been no reports of a relationship between any behavioural factor and the prognosis of CAD in Japan. The purpose of the current study was to investigate behavioural correlations with the prognosis of CAD as a part of the ECGS.

**Methods:**

We examined the mortality and coronary events of 201 men (58 ± 10, 27-86 years) enrolled in the ECGS from 1990 to 1995, who underwent diagnostic coronary angiography and were administered the JCBS and the Japanese version of the Jenkins Activity Survey (JAS) Form C. Their health information after CAG was determined by a review of their medical records and by telephone interviews that took place from 2002 to 2003.

**Results:**

Cardiac events during the follow-up period (7.7 ± 4.2 years) included 13 deaths from CAD, 25 cases of new-onset myocardial infarction, 26 cases of percutaneous coronary intervention, and 19 cases of coronary artery bypass graft surgery. There was no difference in established risk factors between groups with and without cardiac events. Seven factors were extracted by principal component analysis in order to clarify which factors were measured by the JCBS. Stepwise multivariate Cox-hazard regression analysis, in which 9 standard coronary risk factors were forced into the model, showed that Factor 4 from the JCBS (namely, the Japanese spirit of ‘Wa’) was independently associated with coronary events (hazard ratio: 0.21; p = 0.01). By other Cox-hazards regression analyses of coronary events using each set of JAS scores and the JCBS Scale C score instead of Factor 4 as selectable variables, the JAS scores or the JCBS Scale C score were not entered into the models.

**Conclusion:**

The Japanese spirit of ‘Wa’ is a preventive factor against coronary events for Japanese men with CAD.

## Background

Psychosocial factors are recognized as important risk factors for cardiovascular disease (CAD). What has been called the ‘Type A’ behaviour pattern has been described as a coronary-prone behavioural type in Western studies. This pattern is characterized by attributes such as hard-driving effort, striving for achievement, competitiveness, aggressiveness, haste, impatience, restlessness, alertness, uneven bursts of amplitude in speech, and hurried motor movements. Individuals who exhibit this behaviour pattern are usually conscientiously committed to, and often successful in, their occupations [1]. The Secondary Prevention in Uppsala Primary Health Care Project [2] has demonstrated the effectiveness of cognitive behavioural therapy focused on stress management, coping with stress, and reducing experiences of daily stress, time urgency, and hostility in CAD patients. However, the impact and content of risk are thought to differ by geographic region, ethnic origin, and society. In the INTERHEART study [3], general stress and depression in acute myocardial patients had different odds ratios by region and ethnic group. Coronary-disease-prone behaviour in Japan is thought to be characterized by less aggression and a greater tendency to display ‘workaholic’ characteristics than that in Western countries [4].

The Eastern Collaborative Group Study (ECGS) [5] is a multi-centre study in Japan aimed at investigating behavioural correlates of CAD among contemporary Japanese people, beyond the concept of Type A behaviour patterns. In this project, we developed a questionnaire called the Japanese Coronary-prone Behaviour Scale (JCBS), which consists of 122 questions that evaluate 10 behavioural and psychological features: 1) attitude toward one’s job, 2) psychophysiological characteristics, 3) speed and impatience, 4) eating behaviour, 5) style of speech, 6) Japanese mentality, 7) stylistic hostility, 8) emotional hostility, 9) social support, and 10) developmental history [4]. It incorporates items beyond the concept of Type A behaviour patterns, and includes behavioural characteristics specific to Japanese society and cultures. A previous study [6] using the JCBS showed that 9 items from the JCBS, named Scale C, were independently associated with the presence of CAD in patients undergoing coronary angiography (CAG). Scale C represents job-centred lifestyle, social dominance, and suppressed overt Type A behaviour. The external validity of the JCBS Scale C was confirmed by a separate study [7] using different participants.

Although low social support and negative emotions, such as depression, have been shown to aggravate the prognosis of CAD patients in Western countries [8-24], to our knowledge there is no report that clarifies how psychosocial risk factors are related to the prognosis of Japanese CAD patients. The purpose of this study was to investigate behavioural factors predicting the prognosis of Japanese men with established CAD.

## Methods

### Participants

Participants were 201 men of the initial ECGS who had CAD at the index angiogram performed between 1990 and 1995. They were admitted to the hospital one or two days before the CAG. Medical histories and status of coronary risk factors, including hypertension, diabetes mellitus, smoking, and obesity, were obtained on admission day 1 from medical records. Levels of plasma lipids after >14 hours of fasting were assessed on admission day 2. Participants were administered the JCBS and the Japanese version of the Jenkins Activity Survey (JAS) Form C while undergoing diagnostic CAG.

The JAS was developed in an attempt to duplicate the clinical assessment of the Type A behaviour pattern by employing an objective psychometric procedure. The JAS is a self-administered, computer-scored questionnaire that was constructed in an effort to develop a quicker, less expensive, more uniform, and better-calibrated procedure for judging coronary-prone behaviour patterns in large groups of participants [1]. A simple scoring method applied to this test validly identified the behaviour patterns of approximately 72% of a sample of men; the standardized interview was the criterion [1]. Since this initial step toward validation, more sophisticated scaling and scoring procedures have been developed. These have, in turn, been applied retrospectively to a sample of men with coronary heart disease. The JAS has been revised several times and the result is the JAS Form C, which is the version typically used. The JAS yields a composite Type A scale score and three factor-analytically-derived subscales: Speed and impatience (Factor S), Job involvement (Factor J), and Hard-driving and competitive (Factor C).

Participants’ health information after CAG was determined by a review of medical records and telephone interviews from 2002 to 2003. Primary end points were new-onset cardiac events, including cardiac death, angina pectoris, acute myocardial infarction (AMI), percutaneous coronary intervention (PCI), and coronary artery bypass graft (CABG).

This study’s protocol was approved by the Ethical Committee of Aichi Medical University School of Medicine.

### Statistical analysis

The SAS program (SAS Institute, Cary, NC) was used for statistical analysis. Differences in quantitative variables between the two groups were analysed with the Student's *t*-test, and differences in categorical data were analysed with the chi-square test.

Principal component analysis with promax rotations was used to confirm which factors were measured by the JCBS, the questions of which were originally based on behavioural and psychological features, because the abstracted factors correlated with each other: −0.52 for Factors 1 and 6, 0.46 for Factors 3 and 6, 0.51 for Factors 3 and 7, −0.41 for Factors 4 and 5, and 0.44 for Factors 4 and 7. The scree test was used to determine the number of factors to retain in the principal component analysis.

Stepwise multivariate Cox-hazard regression stratified by medical centre was used for evaluating the independent effect of the JCBS factors and standard coronary risks factors. Standard coronary risk factors, including age, body mass index, HDL cholesterol, LDL cholesterol, triglycerides, current smoking habits, severity of coronary stenosis, and history of diabetes and hypertension at the first admission, were forced into this regression model. Continuous values were used for age, body mass index, HDL cholesterol, LDL cholesterol, triglycerides, and the number of coronary artery branches involved; categorical values were used for current smoking habits (i.e. current smoker or not) and presence of diabetes and hypertension. The number of coronary artery branches involved was defined as the number of major coronary arteries or branches with at least one clinically significant stenosis whose luminal narrowing was equal to or more than 50%, or with a lesion that had been the subject of intervention. Clinically significant stenosis in the left main coronary artery was defined as three-vessel stenosis. The presence of hypertension was determined if the subject was under treatment or had equal to or more than 160 mm mercury of systolic blood pressure or equal to or more than 90 mm mercury of diastolic blood pressure on admission. The presence of diabetes was determined if the subject was under treatment or the result of an oral glucose tolerance test met the criteria of diabetes. This analysis was performed after stratifying by hospital because the patients who participated were initially drawn from nine hospitals throughout the country.

## Results

During the follow-up period (7.7 ± 4.2 years), 23 (11%) men died, 13 (6%) of whom died from cardiovascular disease. AMI or angina occurred in 25 (12%) cases, PCI in 26 (13%), and CABG in 19 (9%). Patients with cardiac death, new-onset AMI or angina, PCI, or CABG were counted in the event group. The primary characteristics of patients grouped by cardiac event are shown in Table 1. There were no differences in standard risk factors, JAS scores, or the JCBS Scale C score between groups with and without events.

**Table 1:** **Characteristics of patients grouped by cardiac events**

	**Event (−) (138)**	**Event (+) (63)**	***p*** **value**
Age	59 ± 9	58 ± 10	ns
LDL cholesterol (mg/dl)	131 ± 33	138 ± 38	ns
Triglycerides (mg/dl)	153 ± 91	151 ± 112	ns
HDL cholesterol ( mg/dl)	38 ± 11	37 ± 9	ns
Body mass index (kg/m^2^)	23 ± 2	24 ± 2	ns
Diabetes mellitus (%)	22	19	ns
Hypertension (%)	41	51	ns
Smoking habits (%)	29	32	ns
Number of coronary artery branches involved	2 ± 1	2 ± 1	ns
*JAS Type A score*	−0.2 ± 8.8	−0.6 ± 8.0	ns
Factor S score	−4.3 ± 9.2	−3.6 ± 9.2	ns
Factor J score	−11.1 ± 7.8	−12.9 ± 9.0	ns
Factor H score	−2.0 ± 10.0	−0.2 ± 11.2	ns
JCBS Scale C score	0.24 ± 1.07	0.08 ± 1.07	ns

JAS = Jenkins Activity Survey, HDL = high-density lipoprotein, LDL = low-density lipoprotein, and JCBS = Japanese Coronary-Prone Behaviour Scale.

‘Event’ includes cardiac deaths and new onset of AMI or angina, PCI, and CABG.

Seven factors were extracted by principal component analysis to clarify which factors were measured by the JCBS (Table 2). Since the scree test showed that the smooth decrease of eigenvalues appeared to level off to the right of the 7^th^ plot, we extracted seven factors.

**Table 2:** **Factor loading of each item and factor of the JCBS**

	**Factor 1**	**Factor 2**	**Factor 3**	**Factor 4**	**Factor 5**	**Factor 6**	**Factor 7**
	**(Workaholic)**	**(Speed and impatience)**	**(Social support)**	**(Japanese spirit of ‘Wa’; harmonious groupism)**	**(Happy childhood memories)**	**(Undesirable dietary style)**	**(Independent personality)**
JCBS 7	0.6657	−0.01544	−0.05889	0.03374	−0.06228	−0.05411	0.00081
JCBS 2	0.66939	−0.07549	0.04187	0.02992	0.04105	−0.16959	0.02506
JCBS 4	0.70306	−0.00579	0.17555	0.02065	0.12309	0.02082	−0.1754
JCBS 11	0.69264	0.12182	0.06037	−0.0468	0.16823	0.15507	−0.10799
JCBS 8	0.5968	−0.04097	0.06928	0.02429	−0.04449	0.00047	−0.02594
JCBS 12	0.644	0.1912	0.18932	−0.00729	0.13311	0.04228	0.00158
JCBS 1	0.58644	0.11522	0.17882	−0.1636	0.1866	−0.07635	0.15225
JCBS 5	0.52427	−0.16922	0.24316	−0.02231	0.07559	0.09161	0.17102
JCBS 61	0.45019	0.19799	0.04548	−0.05218	0.22504	−0.18192	0.1344
JCBS 53	0.4999	0.18488	0.26705	−0.21271	0.18524	−0.08068	0.11102
JCBS 9	0.48181	0.10895	0.44359	−0.01062	0.14866	0.07239	0.26366
JCBS 79	0.38811	0.36497	0.06964	0.09301	−0.08315	0.02752	−0.15673
JCBS 59	0.40289	0.28582	0.09261	0.05008	0.16766	−0.18976	0.29137
JCBS 66	0.41422	0.30333	0.36356	0.32523	0.04684	0.18392	0.06059
JCBS 86	0.33111	0.28684	0.158	0.06694	−0.06065	0.12592	0.04206
JCBS 20	0.15001	0.02207	0.00083	0.10009	−0.06369	−0.01951	−0.07811
JCBS 19	−0.33296	0.28941	−0.08421	0.16522	−0.23582	0.09002	0.00166
JCBS 26	−0.07569	0.50125	−0.09204	0.12555	−0.07603	0.22705	−0.17038
JCBS 27	−0.06801	0.51653	0.15822	0.16726	−0.01501	0.25978	0.00435
JCBS 25	0.13943	0.44699	0.09794	0.03393	0.03305	0.02621	0.05655
JCBS 18	−0.05127	0.45742	−0.15281	0.25542	−0.13299	0.08046	−0.02773
JCBS 23	0.22695	0.42874	−0.10483	−0.02407	0.01333	0.03807	0.03509
JCBS 22	0.29365	0.41337	−0.12816	−0.03467	0.03566	0.14669	−0.07292
JCBS 46	−0.00788	0.46771	0.04789	0.20904	−0.14638	0.23808	0.20028
JCBS 30	−0.00656	0.43062	−0.03661	0.15581	0.00808	0.16003	0.12011
JCBS 45	−0.13756	0.43712	−0.18272	0.30337	−0.35336	0.279	−0.07923
JCBS 94	−0.17995	0.4275	−0.16184	0.36802	−0.08971	−0.11907	0.07832
JCBS 44	−0.18733	0.45537	−0.08459	0.40844	−0.43417	0.3293	0.04604
JCBS 52	0.11414	0.38368	0.15322	0.11326	−0.06257	0.12267	−0.16308
JCBS 24	0.31927	0.37819	0.15927	−0.09052	0.15054	−0.12432	0.15631
JCBS 67	0.21982	0.36894	0.09766	−0.10005	0.03008	−0.01498	0.2324
JCBS 42	0.16575	0.4495	0.23697	0.31087	0.03301	0.16148	0.1241
JCBS 95	−0.13882	0.39013	−0.15219	0.25738	−0.18216	−0.14097	0.20038
JCBS 43	−0.20393	0.36447	−0.16696	0.35761	−0.15165	0.08106	−0.20775
JCBS 88	0.03973	0.38072	0.06979	0.23257	0.1357	−0.07148	0.19152
JCBS 81	0.0367	0.34333	0.14592	0.15006	0.12468	−0.05457	0.11338
JCBS 85	0.3001	0.32621	0.12335	−0.0024	0.18982	0.04389	−0.05015
JCBS 17	−0.0318	0.34856	−0.10023	0.21135	−0.20494	0.08518	0.05008
JCBS 97	0.07408	0.33041	0.17667	0.02562	−0.0243	0.30175	0.04942
JCBS 64	0.37515	0.34738	0.22695	0.00772	0.11119	−0.02925	0.11571
JCBS 21	0.09574	0.32233	0.0388	0.20347	−0.04849	−0.02085	0.04544
JCBS 28	0.01861	0.28492	−0.09643	0.04103	−0.08577	0.04365	0.1485
JCBS 14	0.06726	0.24228	−0.07494	−0.05824	0.01332	0.08917	−0.1116
JCBS 77	0.17434	0.29623	−0.08518	0.13654	0.00409	0.01672	0.01262
JCBS 41	−0.08561	0.26419	0.19067	0.06778	−0.05445	0.01807	0.18801
JCBS 36	−0.04394	0.15025	−0.01405	−0.1442	−0.02217	−0.00058	−0.17125
JCBS 90	0.06846	−0.09023	0.64526	−0.0887	0.15949	0.02903	−0.03107
JCBS 91	−0.00203	0.0599	0.58133	0.02309	0.24866	−0.05306	0.06609
JCBS 89	0.29622	−0.08047	0.63338	0.1546	0.15514	0.08962	0.07718
JCBS 96	0.01167	−0.10475	0.55056	−0.18646	0.18498	0.00671	0.05546
JCBS 92	−0.00858	0.01325	0.51722	−0.07883	0.04688	0.02208	−0.09845
JCBS 100	0.2134	−0.06753	0.53014	−0.07476	0.13151	0.08802	−0.04769
JCBS 93	0.17705	−0.08125	0.52357	0.03586	0.12482	0.04661	0.17957
JCBS 99	0.02482	−0.02509	0.4287	−0.13756	0.17436	0.2346	−0.1314
JCBS 87	0.4469	0.00565	0.48002	−0.18349	0.21304	−0.11811	−0.13008
JCBS 56	0.26457	0.11603	0.44431	−0.22329	0.1752	−0.0467	−0.04272
JCBS 48	0.36848	0.15436	0.42301	−0.02913	0.26516	−0.11816	−0.03833
JCBS 57	0.11451	0.2262	0.32766	−0.01572	0.18355	0.02246	−0.02381
JCBS 35	−0.06516	0.04711	0.20655	0.15855	−0.10591	−0.13739	0.07
JCBS 114	0.20497	0.2254	0.29093	0.01448	0.20263	0.03842	0.01016
JCBS 112	−0.07352	−0.00416	−0.22813	−0.0033	0.11824	0.0983	0.02282
JCBS 10	0.29624	0.15131	−0.25948	−0.06458	0.04414	0.20201	−0.0644
JCBS 50	0.08181	0.22404	−0.16142	0.59188	0.01463	0.01825	−0.15079
JCBS 55	−0.02463	0.06693	0.03597	0.56345	−0.01025	0.18295	−0.07541
JCBS 54	−0.01411	0.19381	0.08163	0.54285	−0.17434	0.15345	−0.12387
JCBS 75	−0.06644	0.19038	0.04919	0.53616	−0.21321	0.11027	0.17239
JCBS 49	0.20303	0.0343	0.01538	0.41193	−0.02118	0.06043	−0.22381
JCBS 65	0.28458	0.23561	0.10726	0.4714	−0.0207	0.15894	0.20435
JCBS 47	−0.12532	0.11299	−0.16314	0.47509	−0.13236	0.10697	0.08291
JCBS 78	0.01294	−0.229	0.28982	0.3523	0.0312	−0.02967	−0.06426
JCBS 73	−0.00245	0.1842	−0.0105	0.46701	−0.15905	0.16679	0.23514
JCBS 62	0.2086	0.13547	0.01018	0.37034	0.09751	0.29022	0.06696
JCBS 80	−0.13451	0.34139	−0.27209	0.42403	−0.05445	−0.22374	0.16423
JCBS 68	−0.21354	0.22512	−0.16348	0.34035	0.00638	−0.19272	0.29004
JCBS 29	−0.06933	0.17168	−0.07026	0.26516	−0.10025	0.11571	−0.04141
JCBS 16	0.00698	0.11539	−0.09249	0.13222	−0.06206	−0.00606	0.01106
JCBS 58	0.21747	0.02575	−0.08878	−0.24377	0.22553	−0.18739	0.19668
JCBS 117	0.20268	−0.04706	0.23214	−0.10968	0.72265	−0.11323	0.0042
JCBS 107	0.12388	−0.01471	0.06013	−0.08219	0.59764	−0.02335	0.01895
JCBS 104	0.0409	0.00942	0.21595	−0.09466	0.59397	0.11561	0.10846
JCBS 122	−0.02584	−0.04325	0.19593	0.16139	0.5451	0.01578	0.23426
JCBS 111	0.14297	0.17507	0.20367	−0.03662	0.44208	0.21147	0.0175
JCBS 118	0.27392	0.00152	0.40005	−0.07542	0.43322	0.0299	0.06302
JCBS 121	−0.03252	−0.06697	0.05893	0.06374	0.30071	−0.07693	0.14794
JCBS 72	−0.00927	−0.00648	0.06305	−0.16514	0.25955	−0.1467	−0.05453
JCBS 76	0.21002	0.08095	0.23713	−0.00795	0.21793	0.12333	0.01105
JCBS 102	0.04736	0.18578	0.03297	0.19739	−0.16773	−0.07515	0.18822
JCBS 40	−0.05862	0.22017	0.09371	0.27771	−0.22501	0.10568	0.1409
JCBS 120	−0.07915	0.08305	−0.15092	0.24102	−0.52934	0.15085	0.08448
JCBS 119	−0.10988	0.08002	−0.15743	0.11462	−0.68387	0.10537	0.06965
JCBS 31	0.11864	0.25764	0.13626	0.12208	0.04839	0.52558	−0.0507
JCBS 38	−0.09595	0.13673	−0.12985	0.20347	−0.09528	0.40525	0.03978
JCBS 34	−0.08502	0.14654	−0.0224	0.20199	0.01751	0.37147	0.16665
JCBS 113	−0.0371	0.08721	−0.05023	0.37954	−0.05483	0.38486	0.06201
JCBS 106	0.01366	−0.01016	0.14024	0.17802	−0.01568	0.37374	−0.15778
JCBS 109	0.08942	0.29069	0.11004	0.19584	−0.24665	0.39505	−0.0259
JCBS 74	0.06573	0.25493	−0.00485	0.01242	−0.08814	0.31184	0.30365
JCBS 98	0.03564	0.12135	0.28263	−0.03918	0.07747	0.30424	−0.25118
JCBS 116	0.06314	0.10832	0.19526	0.15951	−0.00241	0.30146	−0.19585
JCBS 110	0.27577	0.15513	0.29255	0.07707	−0.05192	0.29194	0.08385
JCBS 108	0.01961	0.22419	0.05044	0.1961	−0.2488	0.30021	0.06376
JCBS 84	0.03769	−0.12947	0.21008	0.05492	−0.01254	0.26767	−0.16522
JCBS 37	−0.07552	0.12013	0.00512	0.13458	0.00274	0.24435	−0.09291
JCBS 105	−0.06563	0.06894	−0.08361	−0.02213	−0.02628	0.22403	−0.01352
JCBS 33	−0.04418	0.0593	0.05493	0.09348	−0.17229	0.21418	0.03581
JCBS 15	−0.01361	0.02728	−0.09501	0.1109	0.03791	0.17647	−0.08715
JCBS 39	0.21319	0.05671	0.18596	0.04633	0.12117	−0.16607	0.03272
JCBS 32	0.20133	0.11511	0.08742	0.0111	0.15387	−0.48421	0.02026
JCBS 83	−0.0253	0.24312	−0.11168	0.20914	0.001	−0.06525	0.50581
JCBS 71	−0.16415	0.36606	0.01562	0.30324	−0.09696	−0.00908	0.51968
JCBS 82	0.18439	0.19497	0.19233	−0.12583	0.11283	−0.13636	0.38774
JCBS 60	0.25375	0.02935	0.01432	0.08277	0.10098	−0.13155	0.36704
JCBS 69	0.23954	0.39511	0.28687	0.08939	0.07815	0.01352	0.39382
JCBS 70	0.40468	0.30484	0.20857	−0.09125	0.22714	−0.10205	0.37923
JCBS 115	0.02126	0.07131	0.11341	−0.08057	0.22347	−0.14141	0.29409
JCBS 103	0.12435	−0.00423	0.0897	−0.0369	0.14394	−0.10055	0.25367
JCBS 101	−0.02303	0.14205	0.03216	0.23899	−0.16747	−0.06186	0.26872
JCBS 51	0.31593	0.14067	0.32535	0.10465	0.23092	−0.18426	−0.20066
JCBS 6	−0.13144	0.10375	0.05389	0.11223	−0.11872	−0.17435	−0.27017
JCBS 3	0.26648	0.04012	0.19152	0.11079	0.03446	−0.10922	−0.31547
JCBS 13	0.26072	0.20354	0.21544	0.10559	0.04976	−0.09524	−0.38319
JCBS 63	0.08246	0.08367	0.03838	−0.01768	0.14894	−0.08197	−0.43729
Un-weighted explained variance	6.1660478	5.4356435	4.6859952	4.0632515	3.5970369	3.2892574	3.4035135

JCBS = Japanese Coronary-prone Behaviour Scale.

The results of Cox-hazards regression analysis of coronary events with JCBS factors and standard coronary risks are shown in Table 3. Nine standard coronary risk factors were forced into the model, and HDL cholesterol was identified as a significant predictor of the recurrence of cardiovascular events. In that model, Factor 4 on the JCBS was extracted as a significant and independent predictor. By other similar Cox-hazards regression analyses of coronary events using each set of JAS scores and the JCBS Scale C score instead of Factor 4 as selectable variables and after 9 standard coronary risk factors were forced into the model, the JAS scores or the JCBS Scale C score were not entered into the models (not shown).

**Table 3:** **Cox hazards regression analysis of coronary events with JCBS factors and standard coronary risks**

**Variable**	**Hazard ratio (95% CI)**	***p*** **value**
*Forced entry*		
Age	0.99 (0.90-1.09)	0.85
LDL cholesterol	0.98 (0.95-1.01)	0.14
Triglycerides	1.00 (1.00-1.01)	0.49
HDL cholesterol	0.86 (0.76-0.98)	0.02
Body mass index	1.12 (0.79-1.59)	0.52
Diabetes mellitus	0.53 (0.06-4.41)	0.55
Hypertension	0.20 (0.04-1.04)	0.06
Smoking habits	1.50 (0.26-8.52)	0.67
Number of coronary artery branches involved	2.61 (0.86-7.84)	0.09
*Selected*		
Factor 4	0.21 (0.06-0.71)	0.01

JAS = Jenkins Activity Survey, HDL = high-density lipoprotein, LDL = low-density lipoprotein, and JCBS = Japanese Coronary-prone Behaviour Scale.

‘Event’ includes cardiac deaths and new-onset of AMI or angina, PCI, and CABG.

## Discussion

This follow-up study of Japanese men with established CAD showed that the fourth factor of the JCBS had a protective effect against cardiovascular events. This is the first follow-up study in which the relationship between behavioural factors and CAD prognosis was examined among Japanese male patients.

Contrary to findings in other countries, a prospective analysis in a Japanese population shows that lower levels of impatience were associated with a 1.4-fold higher multivariable-adjusted risk of incidence of coronary heart disease and a 1.5-fold higher multivariable-adjusted risk of incidence of myocardial infarction and non-fatal coronary disease among Japanese men [7]. In our study, neither JAS Type A score nor any other subscale score was associated with CAD prognosis. However, our participants had job-centred lifestyles, social dominance, and suppressed overt Type A behaviour in the previous JCBS study [5], and did not have any difference in JAS Factor S scores (Speed and impatience scale score) from male participants without CAD. This could be caused by the difference in measurement of speed and impatience, since the JAS Factor S score is calculated from answers to several items, but impatience in the Japan Public Health Centre-based prospective Study [7] was assessed from a single item answer.

The fourth factor of the JCBS contains questions about the Japanese mentality, such as ‘You are always worried about what others think of you’ (JC50), ‘You put a good face on your elders and betters’ (JC55), ‘You often sacrifice yourself for others (JC75)’, ‘You think much of the public image’ (JC49), and so on. We called the fourth factor the Japanese spirit of ‘Wa’ (‘harmonious groupism’), thought to be a Japanese traditional attitude used to keep order in hierarchically organized social relationships in communities and groups. ‘Wa’ represents a way of living that integrates a person into his or her community or group.

Some prospective studies in Western countries have implicated emotional distress, depression [8-13], and lack of social support [14-24] as risk factors for CAD prognosis. In Japan, the Osaka Acute Coronary Insufficiency Study showed that symptoms of depression were related to cardiovascular events [25,26]. The Shizuoka elderly cohort study showed that more positive individual perceptions of community cohesion are associated with reduced risks of cardiovascular disease, pulmonary disease, and all other causes of mortality [27]. In our study, the third factor of the JCBS was thought to be an index of social support because it contains items about family or friends, such as ‘You often talk together with your family’ (JC90), ‘Your family listens to your problems’ (JC91), ‘Your friends listen to your problems’ (JC92), and so on. It was not a significant factor. This could mean that the third factor of the JCBS was not the same as the structural or functional social support [28] for which a significant relationship to CAD has been reported.

This study has some limitations. First, the mechanism that explains the relationship between CAD prognosis and the Japanese spirit of ‘Wa’ is not well known. Chronic stress and affective disorders are thought to have pathophysiological mechanisms that promote atherosclerosis [29]. The Japanese spirit of ‘Wa’ might have similar mechanisms. Second, nine medical centres collaborated in this study, which included community-based and intensive medical centres in different regions. This could have affected the accessibility of follow-up data. We analysed data stratified by centres to decrease this bias.

## Conclusion

The Japanese spirit of ‘Wa’ may have protective effects against cardiovascular events among Japanese men with established CAD.

## Abbreviations

AMI: Acute myocardial infarction
CABG: Coronary artery bypass graft
CAD: Cardiovascular disease
CAG: Coronary angiography
ECGS: Eastern Collaborative Group Study
JAS: Jenkins Activity Survey
JCBS: Japanese Coronary-prone Behavior Scale
PCI: Percutaneous coronary intervention
